# Data enhancement for co-morbidity measurement among patients referred for sleep diagnostic testing: an observational study

**DOI:** 10.1186/1471-2288-9-50

**Published:** 2009-07-15

**Authors:** Paul E Ronksley, Willis H Tsai, Hude Quan, Peter Faris, Brenda R Hemmelgarn

**Affiliations:** 1 Department of Community Health Sciences, Faculty of Medicine, University of Calgary, Calgary, Canada; 2 Department of Medicine, Faculty of Medicine, University of Calgary, Calgary, Canada

## Abstract

**Background:**

Observational outcome studies of patients with obstructive sleep apnea (OSA) require adjustment for co-morbidity to produce valid results. The aim of this study was to evaluate whether the combination of administrative data and self-reported data provided a more complete estimate of co-morbidity among patients referred for sleep diagnostic testing.

**Methods:**

A retrospective observational study of 2149 patients referred for sleep diagnostic testing in Calgary, Canada. Self-reported co-morbidity was obtained with a questionnaire; administrative data and validated algorithms (when available) were also used to define the presence of these co-morbid conditions within a two-year period prior to sleep testing.

**Results:**

Patient self-report of co-morbid conditions had varying levels of agreement with those derived from administrative data, ranging from substantial agreement for diabetes (κ = 0.79) to poor agreement for cardiac arrhythmia (κ = 0.14). The enhanced measure of co-morbidity using either self-report or administrative data had face validity, and provided clinically meaningful trends in the prevalence of co-morbidity among this population.

**Conclusion:**

An enhanced measure of co-morbidity using self-report and administrative data can provide a more complete measure of the co-morbidity among patients with OSA when agreement between the two sources is poor. This methodology will aid in the adjustment of these coexisting conditions in observational studies in this area.

## Background

Obstructive Sleep Apnea (OSA) is a disorder characterized by periods of cessation of breathing during sleep with intermittent hypoxemia and sleep fragmentation. Population-based studies estimate the prevalence of OSA to be approximately 3 to 7% for adult males and 2 to 5% for adult females in the general population [1-4]. Furthermore, patients with OSA commonly have other medical conditions, including hypertension, stroke, cardiovascular disease, and cardiac arrhythmia [5-14].

Given the increased morbidity associated with OSA, observational studies of patients with OSA must also adjust for these co-morbid conditions to determine the independent effect of OSA on the outcomes of interest. The use of self-reported data through questionnaires or interviews is a common method of determining the presence of co-morbid conditions due to its efficiency and relative low cost. However, the reliability and accuracy of this data is questionable [15-21]. In addition the validity of self-reported conditions, using medical records as the gold standard, varies depending on the medical conditions in question and the target population under investigation [15-21].

Administrative data is another source from which to determine the presence of co-morbid conditions. While agreement between self-reported medical conditions and that obtained from administrative databases also varies [22-27], combining self-reported clinical data with that obtained from administrative data has been proposed as a method to increase the completeness and accuracy of co-morbid conditions [28-30]. This enhanced measure of co-morbidity has been undertaken and shown to provide a valid assessment for patients with coronary heart disease and those undergoing coronary artery bypass graft surgery [31-33]. Previous studies have assessed co-morbidity in OSA patients in the years prior to diagnosis [34,35]. However, many of these studies have relied on administrative records alone to determine co-morbidity. This source alone may result in an underestimate of co-morbidity within these populations. Given the importance of co-morbidity in observational studies of OSA patients, and the limited information in the literature on studies combining data sources to measure co-morbidity, the purpose of this study was to evaluate whether the combination of administrative data and self-reported data provided a more complete estimate of co-morbidity among patients referred for sleep diagnostic testing.

## Methods

### Study Design

This project is part of a larger retrospective study investigating health care utilization among patients with OSA. We included all adult patients (> 18 years old) referred for sleep diagnostic testing at either a hospital location in Calgary, Alberta, or private home care facilities within the Calgary Health Region between July 2005 to August 2007. Virtually all sleep diagnostic testing for the city of Calgary and surrounding areas (population of approximately 1.3 million) is conducted in these facilities. All patients who underwent polysomnography (PSG) or ambulatory monitoring for the presence of OSA were invited to participate in the study. We excluded non-Alberta residents, patients previously diagnosed with OSA, and those referred but did not undergo diagnostic testing.

### Obstructive Sleep Apnea

We used polysomnography (PSG) and ambulatory monitoring to identify OSA within participants. Although PSG is considered the 'gold standard' diagnostic test for OSA, an ambulatory monitoring device has proven to have excellent agreement, sensitivity and specificity with PSG [36]. In addition, the use of ambulatory monitoring has been validated as a clinical management tool [37,38].

We stratified patients by OSA severity, based on their sleep test results, using the respiratory disturbance index (RDI). The RDI was defined as the number of apneas and hypopneas per hour of sleep. Apnea was defined as a cessation of airflow for at least 10 seconds. Hypopnea was defined as an abnormal respiratory event lasting 10 seconds or more, with at least a 30% reduction in thorocoabdominal movement or airflow compared to baseline, and associated with at least a 4% oxygen desaturation. OSA severity categories included: no OSA (RDI <5 event/hr), mild OSA (RDI 5–14.9 events/hr), moderate OSA (RDI 15–29.9 events/hr) and severe OSA (RDI ≥ 30 events/hr). This classification system is well accepted in both clinical practice and within the medical literature [39,40]. The date of the sleep study was used to define the index date.

### Determination of Co-morbidities and Clinical Characteristics from Surveys

Baseline clinical and demographic information was collected for all participants prior to sleep diagnostic testing. This included: age, sex, height, weight, body mass index (BMI), neck circumference, and postal code. Each participant also completed the Epworth Sleepiness Scale (ESS) [41], a self-administered questionnaire that provides a measure of daytime sleepiness. Co-morbidity was determined through the use of a questionnaire administered by trained personnel within the clinics, and patients were asked to self-report the presence of nine specific co-morbidities including hypertension, asthma, depression, cardiac arrhythmia, myocardial infarction, chronic obstructive pulmonary disease (COPD), diabetes, heart failure, and stroke. Patients were also required to provide a list of their current medications at the time of the survey. This study was approved by the Ethics Review Board of the University of Calgary, and patients gave written informed consent to participate in the study.

### Determination of Co-morbidities from Administrative Data Sources

Using the patient's unique Provincial Health Number (PHN), the cohort was linked to two Alberta Health and Wellness administrative databases, the hospitalization discharge database, and the physician claims database. For each patient, all hospitalization and physician claims information was obtained for a two-year period prior to sleep diagnostic testing.

The hospital inpatient data source contains details regarding hospitalizations including admission date, discharge date, length of stay, 25 diagnostic codes (ICD-10), and 10 procedure codes for each admission. The physician claims registry contains information on physician services including dates and location of the visits, diagnostic codes (ICD-9-CM), provider specialty, and include the majority of residents in the province except a small proportion of special population groups (i.e. members of the Armed Forces, Royal Canadian Mounted Police (RCMP), and federal inmates – accounting for approximately 1% of the total population) [42].

Co-morbid conditions were identified within the Alberta Health and Wellness administrative databases using the International Classification of Diseases (ICD-9-CM and ICD-10) definitions for the nine specific co-morbidities. When available, validated algorithms were used to define each co-morbid condition (Table 1) [43-48]. These algorithms were further supplemented by the ICD-10 coding scheme developed by Quan et al. [49]. For co-morbidities that did not have validated algorithms (specifically COPD, depression and cardiac arrhythmia), ICD-9-CM and ICD-10 diagnostic codes were identified within the ICD-9-CM and ICD-10 manuals [50,51]. Within the administrative datasets, the condition was considered present if the algorithm defining the condition was satisfied. For example, diabetes was considered present if there were two or more separate diagnostic codes identifying diabetes within the physician claims or one or more hospitalization diagnostic codes identifying diabetes within the a two year period [44]. Co-morbidities that did not have a validated algorithm (depression, COPD and cardiac arrhythmia) were considered present if at least one diagnostic code recorded for the condition within either the physician claims data or hospitalization data was recorded within the two-year period prior to the index date. All 3 diagnostic coding fields were used within the physician claims data and all 25 diagnostic codes within inpatient hospitalization data. We used diagnostic type indicators in this data source to restrict conditions to only those present prior to admission and therefore excluded any condition that developed while staying in hospital.

**Table 1:** ICD-9-CM and ICD-10 Codes to Define Co-morbidity Among Patients Referred for Sleep Diagnostic Testing

Co-morbidities	Authors	Algorithm	ICD-10 diagnostic codes	ICD-9-CM diagnostic codes	Sensitivity	Specificity	PPV
Hypertension (with and without complication)	Tu et al.[43]	2 physician claims in 3 years	I10.x, I11.x–I13.x, I15.x	401.x, 402.x–405.x	73%	95%	87%

Diabetes (with and without complication)	Hux et al.[44]	1 hospitalization or 2 physician claims in 2 years	E10.0-E10.9, E11.0-E11.9, E12.0-E12.9, E13.0-E13.9, E14.0-E14.9	250.0–250.9	86%	97%	80%

Asthma	Huzel et al.[45]	1 or more physician claims in 2 years	J45.0, J45.1, J45.8, J45.9	490.0, 491.0, 492.0, 493.0	50.9%	98.1%	NR

Myocardial Infarction	Austin et al.[46]	Primary discharge diagnosis of AMI in hospitalization database	I21.x, I22.x, I25.2	410.x	88.8%	92.8%	88.5%

Congestive Heart Failure	Lee et al.[47]	Primary discharge diagnosis of CHF in hospitalization database	I09.9, I11.0, I13.0, I13.2, I25.5, I42.0, I42.5–42.9, I43.x, I50.x, P29.0	428.x	NR	NR	94.3%

Cerebrovascular Accident/Transient Ischemic Attack	Kokotailo and Hill[48]	Primary discharge diagnosis of stroke in hospitalization database	H34.1, I63.x, I64.x, I61.x, I60.x, G45.x	362.3, 430.x, 431.x, 433.x1, 434.x1, 435.x, 436	67%	97%	84%

COPD	No Validated Algorithm		J44	491.21, 493.2, 496	NR	NR	NR

Depression	No Validated Algorithm		F20.4, F31.3-F31.5, F32.x, F33.x, F34.1, F41.2, F43.2	296.2, 296.3, 296.5, 300.4, 309.x, 311	NR	NR	NR

Cardiac Arrhythmia	No Validated Algorithm		I44.1-I44.3, I45.6, I45.9, I47.x-I49.x, R00.0, R00.1, R00.8, T82.1, Z45.0, Z95.0	426.0, 426.1, 426.7, 426.9, 426.10, 426.12, 427.0–427.4, 427.6–427.9, 785.0, 996.01, 996.04, V45.0, V53.3	NR	NR	NR

Abbreviations: NR = Not Reported

### Analysis

Patient characteristics were described using mean and standard deviation for normally distributed variables. In cases of highly skewed or non-normal distributions, the median and the inter-quartile range (IQR) were reported. Means and proportions were compared using analysis of variance and chi-square tests respectively. In addition, proportions of patients presenting with specific co-morbidities, identified in the questionnaire, were calculated.

To assess the agreement between self-reported co-morbidity and administrative databases, we calculated the proportion of subjects with each co-morbid condition based on: self-report only, administrative data sources only, both self-report and administrative data, and either self-report or administrative data. To evaluate consistency between self-report and administrative data the Kappa (κ) statistic and 95% confidence intervals were calculated. The Kappa statistic is an index of the degree of agreement between two raters, and can be thought of as the chance-corrected proportional agreement; possible values range from +1 (perfect agreement) to 0 (no agreement above that expected by chance). Kappa values were defined as: < 0.40 as poor or fair agreement, 0.40–0.60 as moderate agreement, 0.61–0.80 as substantial agreement, and 0.81–1.00 as almost perfect agreement [52].

In addition, the McNemar's test of paired proportions was determined. This is a statistical procedure to compare two dependent or correlated proportions, and is a test of marginal homogeneity that compares agreement between discordant pairs. A statistically significant McNemar's test would indicate a difference between the proportions compared. Finally to assess the validity of the enhanced measures of co-morbidity an analysis was also performed in which patients were stratified by severity of OSA to determine trends in the prevalence of the co-morbid conditions. All statistical analysis was conducted using STATA 10.0 software (Statacorp, College Station, Texas).

## Results

### Study Participants

**Figure 1:**
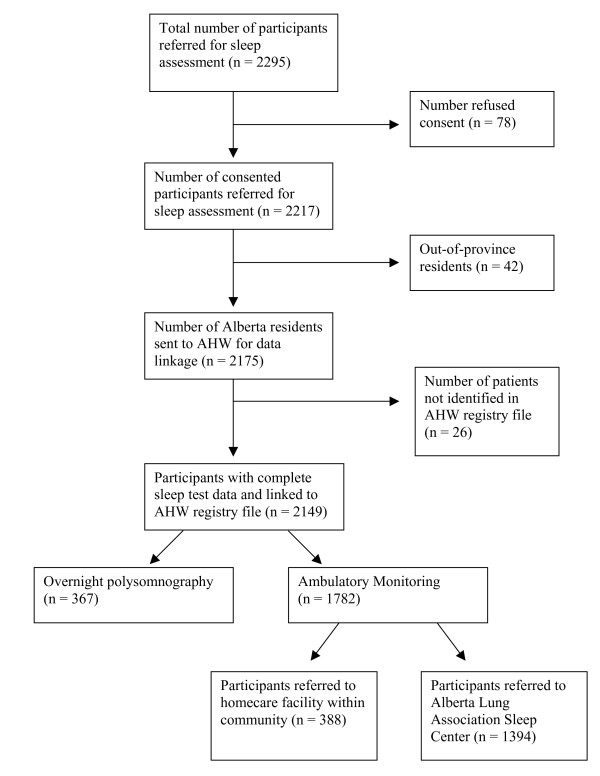
**Patient Flow Diagram**.

From July 2005 to August 2007, 2295 patients were referred for sleep diagnostic testing, of whom 78 (3.4%) patients refused to participate and 42 (1.8%) patients were from out of province and were therefore excluded. Of the remaining 2175 patients, 26 (1.2%) were excluded because they were not present in the Alberta Health and Wellness registry file, for a final study population size of 2149 (Figure 1). Within this study population, 367 patients underwent full overnight polysomnography and the remainder (n = 1782) had ambulatory monitoring either through a private home care facility (n = 388) or through the Alberta Lung Association Sleep Clinic (n = 1394). From the study cohort, 432 (20.1%) patients were identified as having no OSA, 738 (34.3%) with mild OSA, 443 (20.6%) with moderate OSA and 536 (24.9%) with severe OSA. Descriptive characteristics of study subjects, by OSA severity, are presented in Table 2. Overall patients with severe OSA were more likely to be male, older and have a higher Epworth Sleepiness Score compared to subjects with lesser degrees of OSA.

**Table 2:** Patient Characteristics

	All (n = 2149)	No OSA (n = 432)	Mild OSA (n = 738)	Moderate OSA (n = 443)	Severe OSA (n = 536)	p-value*
Male n (%)	1346 (62.6)	197 (45.6)	463 (62.7)	281 (63.4)	405 (75.6)	<0.001
Age, yrs mean (SD)	50.1 (12.9)	44.0 (12.9)	50.0 (12.5)	52.8 (12.5)	53.0 (11.9)	<0.001
BMI, kg/m^2 ^median (IQR)	31.3 (27.3, 36.6)	27.8 (24.9, 32.2)	30.6 (27.2, 35.4)	32.0 (28.1, 36.8)	34.5 (30.4, 39.8)	<0.001
Epworth Sleepiness Score, mean (SD)	11.3 (5.4)	10.9 (5.1)	10.7 (5.3)	11.4 (5.4)	12.4 (5.5)	<0.001
Current Smoker, n (%)	354 (16.5)	89 (20.6)	116 (15.7)	60 (13.5)	89 (16.6)	0.111

Abbreviations: BMI = body mass index; IQR = inter-quartile range; SD = standard deviation

* Chi-squared test, analysis of variance (3 degrees of freedom)

### Comparison of Co-Morbidity Determined by Self-Report and Administrative Data Algorithms

Table 3 presents the prevalence and agreement for co-morbidities determined by self-report and administrative data. The most prevalent condition in both self-report and administrative data was hypertension and depression, with 35.1% and 27.0% of subjects referred for sleep testing self-reporting the presence of these conditions respectively. The proportions based on self-report and administrative algorithms differed significantly (McNemar's p value < 0.05) for all conditions except depression and COPD. There was substantial agreement between self-report and administrative algorithms for diabetes, with a κ = 0.79. There was good agreement for hypertension (κ = 0.60), depression (κ = 0.50) and asthma (κ = 0.49). However COPD, heart failure, myocardial infarction, stroke and cardiac arrhythmia all demonstrated poor agreement. Of note, there was a large discrepancy between self-report and administrative data for the presence of cardiac arrhythmia (5.7% vs. 30.4%).

**Table 3:** Agreement between Self-reported Co-Morbidity and Administrative Measure of Co-Morbidity

Co-morbidity	Self-Report	Administrative Algorithms	Present in Both Data Sources	Present in Either Data Source	Kappa^†^ (95% CI)	McNemar's^††^ p-value
Hypertension n (%)	754 (35.1)	714 (33.2)	539 (25.1)	929 (43.2)	0.60 (0.52, 0.63)	0.0428
Diabetes n (%)	289 (13.4)	238 (11.1)	214 (10.0)	313 (14.6)	0.79 (0.75, 0.83)	<0.001
Asthma n (%)	358 (16.7)	247 (11.5)	168 (7.8)	437 (20.3)	0.49 (0.43, 0.54)	<0.001
Myocardial Infarction n (%)	194 (9.0)	53 (2.5)	37 (1.7)	210 (9.8)	0.27 (0.20, 0.35)	<0.001
Heart Failure n (%)	72 (3.4)	29 (1.3)	15 (0.7)	86 (4.0)	0.28 (0.17, 0.40)	<0.001
Stroke n (%)	62 (2.9)	9 (0.4)	8 (0.4)	63 (2.9)	0.22 (0.09, 0.35)	<0.001

No defined Algorithm	Self-Report	No defined Algorithm*	Both	Either	Kappa (95% CI)	McNemar's p value

Depression n (%)	581 (27.0)	573 (26.7)	364 (16.9)	790 (36.8)	0.50 (0.45, 0.54)	0.6983
Cardiac Arrhythmia n (%)	123 (5.7)	654 (30.4)	86 (4.0)	691 (32.2)	0.14 (0.10, 0.17)	<0.001
COPD n (%)	67 (3.1)	77 (3.6)	24 (1.1)	120 (5.6)	0.31 (0.21, 0.41)	0.3074

* At least one physician claim or hospitalization diagnosis in 2 years.

^† ^The Kappa statistic is an index of the degree of agreement between two raters.

^†† ^McNemar's test of paired proportions is a test of marginal homogeneity that compares the agreement between discordant pairs.

When "both" self-reported and administrative measures of co-morbidity were required to define each condition, proportions for all nine conditions were much lower when compared to a definition that required "either" self-report or administrative measure. For example, the proportion of patients with hypertension was 25.1% when "both" were used and 43.2% when "either" was used.

### Co-Morbidity Measurement by OSA Severity

The prevalence of each of the nine conditions determined by self-report and administrative algorithms, stratified by OSA severity, are presented in Table 4. Based on self-report alone, the prevalence of hypertension, diabetes, and myocardial infarction increased as OSA severity increased. When using the administrative algorithms, a similar trend was observed for hypertension, diabetes and stroke. Table 5 depicts the "enhanced" co-morbidities based on a combination of either self-report or administrative data. The prevalence of hypertension, diabetes and myocardial infarction all increased with increasing OSA severity (p < 0.001).

**Table 4:** Self-reported Co-Morbidity and Administrative Measure of Co-Morbidity Stratified by OSA Severity

	Self-Report				Administrative Algorithms			
Co-morbidity	No OSA (n = 432)	Mild OSA (n = 738)	Moderate OSA (n = 443)	Severe OSA (n = 536)	No OSA (n = 432)	Mild OSA (n = 738)	Moderate OSA (n = 443)	Severe OSA (n = 536)

Hypertension	22.7%	29.1%	42.2%	47.4%	19.2%	27.8%	41.3%	45.3%
Depression	30.1%	28.0%	28.4%	22.0%	29.4%	27.2%	27.8%	22.3%
Diabetes	7.6%	10.6%	11.5%	23.7%	6.7%	8.3%	9.5%	19.8%
Asthma	19.7%	17.1%	17.8%	12.7%	12.3%	10.6%	14.2%	9.9%
COPD	3.2%	2.7%	4.3%	2.6%	3.5%	3.3%	3.4%	4.3%
Myocardial Infarction	4.2%	8.5%	11.1%	11.9%	0.9%	2.0%	1.8%	4.9%
Heart Failure	1.4%	3.1%	2.9%	5.6%	0.5%	1.2%	1.1%	2.4%
Stroke	2.3%	2.2%	2.9%	4.3%	0%	0.1%	0.7%	0.9%
Cardiac Arrhythmia	4.2%	6.0%	6.5%	6.0%	23.4%	28.7%	35.0%	34.7%

**Table 5:** Enhanced Measure of Co-Morbidity using Either Self-Report or Administrative Databases Stratified by OSA Severity

	Either Self-Report or Administrative Database (Enhanced)				
Co-morbidity	No OSA (n = 432)	Mild OSA (n = 738)	Moderate OSA (n = 443)	Severe OSA (n = 536)	p-value*

Hypertension	28.0%	36.3%	51.0%	58.6%	<0.001
Depression	40.3%	37.5%	38.1%	31.7%	0.12
Diabetes	9.3%	11.9%	12.6%	24.1%	<0.001
Asthma	20.8%	21.3%	21.7%	17.5%	0.41
COPD	5.6%	4.7%	6.8%	5.8%	0.56
Myocardial Infarction	4.6%	8.9%	11.5%	13.6%	<0.001
Heart Failure	1.6%	3.5%	3.4%	7.1%	0.09
Stroke	2.3%	2.2%	3.2%	4.3%	0.15
Cardiac Arrhythmia	24.8%	31.0%	36.6%	36.0%	0.08

* Chi-squared test (3 degrees of freedom)

## Discussion

In this large cohort of patients referred for sleep testing we determined that patient self-report of nine co-morbid conditions had varying levels of agreement with that derived from administrative data. Specifically, agreement was highest for diabetes and hypertension, and lowest for cardiac arrhythmia and stroke. An enhanced measure of co-morbidity using either self-report or administrative data demonstrated face validity and clinically meaningful trends of increasing prevalence by OSA severity. These results suggest that when agreement between data sources is poor, a combination of sources should be used when defining co-morbidity in OSA patients, as use of either source alone may result in an underestimate of the prevalence of these conditions. Specifically, using "either" self-report or administrative measure will increase the sensitivity of the estimate of co-morbidity.

We found that among patients referred for sleep testing, self-report of diabetes and hypertension had the highest agreement with administrative data derived definitions for these conditions. These findings are similar to those reported based on administrative data and survey data from an adult sample extracted from the Canadian Community Health Survey (CCHS) in Manitoba, Canada. Agreement between the two sources was highest for diabetes (κ > 0.70) and hypertension (κ > 0.50), and lowest for non-specific heart disease (κ = 0.38) [30]. Cricelli et al. also found good agreement between self-reported diabetes and hypertension and administrative data sources [25]. The consistency of self-reported and administrative data for these two conditions likely occurs because these conditions have clear objective criteria for diagnosis and require ongoing medical treatment. Agreement between self-reported measures of chronic disease and administrative data is dependent on the condition specifically [30].

We found very poor agreement between self-report and administrative data for the presence of cardiac arrhythmia and stroke. Underreporting of cardiac arrhythmia likely occurred because respondents are not aware of the diagnoses, or lack of familiarity with this medical term found on the self-report questionnaire [30]. Though cardiac arrhythmia is common in patients with OSA with prevalence values ranging from 35–48% [13,14], accurate self-reporting is more likely to occur for conditions that require frequent contacts with a health professional; cardiac arrhythmia is not one of these conditions. The enhanced definition of cardiac arrhythmia in our study is similar to the known prevalence in this population, and thus is likely to be an accurate reflection of the prevalence of this co-morbidity within the cohort (32.2%). The poor agreement between the two sources for stroke was also an interesting finding. We speculate that the discrepancies between administrative data and self-report for identifying stroke are due to the lower sensitivity of the administrative algorithm (67%), thus underestimating the true prevalence within this source. Again, the combination of either source likely provides a more accurate representation of stroke prevalence in this clinical population.

The measure of co-morbidity using the enhanced combination of data sources found that as OSA severity increased, the prevalence of hypertension, diabetes, and myocardial infarction also increased. This dose-response relationship for these specific conditions by OSA severity has been documented in previous studies [5,10,53-55] and provides support for the face validity of our enhanced measures of co-morbidity.

The results of our study should be interpreted in context of the study limitations. First, for three of the conditions of interest (depression, cardiac arrhythmia, and COPD), validated administrative algorithms were unavailable. Using an algorithm of at least one physician claim or hospitalization in a two-year period may have resulted in some misclassification and an over-reporting of these conditions. Secondly, we did not have a gold standard to determine whether the enhanced measures are more valid than a single data source alone. However, the increasing prevalence of conditions by OSA severity, consistent with that in the literature, does provide evidence of face validity. Finally, our study was limited to a single geographic region (Calgary Health Region) and only included patients referred for sleep diagnostic testing. These patients likely represent those with more severe morbidity and will limit the generalizability of these results to other clinic-based sleep cohorts in North America.

## Conclusion

We found that administrative data in combination with survey data has the potential to create a more complete measure of the co-morbidity among patients referred for sleep diagnostic testing, particularly when agreement between survey and administrative data is poor. Given the resources required to obtain clinical data, use of data enhancement with administrative data may be valuable to other researchers. Although, future studies are required to validate co-morbidities based on data enhancement, these results suggest that this methodology can aid in the adjustment of these coexisting conditions in observational studies in this area.

## Competing interests

The authors declare that they have no competing interests.

## Authors' contributions

PER Study design, data analysis, and manuscript preparation. WHT Study design, interpretation of results, manuscript preparation. HQ: Interpretation of results, manuscript preparation and critical review. PF: Data analysis, interpretation of results, and critical review. BRH: Study design, data analysis, and manuscript preparation. All authors read and approved the final manuscript.

## Pre-publication history

The pre-publication history for this paper can be accessed here:

http://www.biomedcentral.com/1471-2288/9/50/prepub
